# Implanted Microsensor Continuous IOP Telemetry Suggests Gaze and Eyelid Closure Effects on IOP—A Preliminary Study

**DOI:** 10.1167/iovs.62.6.8

**Published:** 2021-05-06

**Authors:** Jacqueline J. O. N. van den Bosch, Vincenzo Pennisi, Azzurra Invernizzi, Kaweh Mansouri, Robert N. Weinreb, Hagen Thieme, Michael B. Hoffmann, Lars Choritz

**Affiliations:** 1 Department of Ophthalmology, University Hospital Magdeburg, Germany; 2 Implandata Ophthalmic Products GmbH, Hannover, Germany; 3 Laboratory for Experimental Ophthalmology, University of Groningen, University Medical Center Groningen, Groningen, The Netherlands; 4 Cognitive Neuroscience Center, Department of Biomedical Sciences of Cells & Systems, University Medical Center Groningen, Groningen, The Netherlands; 5 Glaucoma Research Center, Montchoisi Clinic, Swiss Visio, Lausanne, Switzerland; 6 Department of Ophthalmology, University of Colorado, Denver, Colorado, United States; 7 Hamilton Glaucoma Center, Viterbi Family Department of Ophthalmology and Shiley Eye Institute, University of California, San Diego, La Jolla, California, United States; 8 Center for Behavioral Brain Sciences, Magdeburg, Germany

**Keywords:** continuous, intraocular pressure, telemetry, gaze direction, eyelid closure

## Abstract

**Purpose:**

To explore the effect of gaze direction and eyelid closure on intraocular pressure (IOP).

**Methods:**

Eleven patients with primary open-angle glaucoma previously implanted with a telemetric IOP sensor were instructed to view eight equally-spaced fixation targets each at three eccentricities (10°, 20°, and 25°). Nine patients also performed eyelid closure. IOP was recorded via an external antenna placed around the study eye. Differences of mean IOP between consecutive gaze positions were calculated. Furthermore, the effect of eyelid closure on gaze-dependent IOP was assessed.

**Results:**

The maximum IOP increase was observed at 25° superior gaze (mean ± SD: 4.4 ± 4.9 mm Hg) and maximum decrease at 25° inferonasal gaze (−1.6 ± 0.8 mm Hg). There was a significant interaction between gaze direction and eccentricity (*P* = 0.003). Post-hoc tests confirmed significant decreases inferonasally for all eccentricities (mean ± SEM: 10°: −0.7 ± 0.2, *P* = 0.007; 20°: −1.1 ± 0.2, *P* = 0.006; and 25°: −1.6 ± 0.2, *P* = 0.006). Eight of 11 eyes showed significant IOP differences between superior and inferonasal gaze at 25°. IOP decreased during eyelid closure, which was significantly lower than downgaze at 25° (mean ± SEM: −2.1 ± 0.3 mm Hg vs. −0.7 ± 0.2 mm Hg, *P* = 0.014).

**Conclusions:**

Our data suggest that IOP varies reproducibly with gaze direction, albeit with patient variability. IOP generally increased in upgaze but decreased in inferonasal gaze and on eyelid closure. Future studies should investigate the patient variability and IOP dynamics.

Intraocular pressure (IOP), the only known treatable risk factor of glaucoma,^1^ typically is measured with Goldmann applanation tonometry (GAT). However, GAT is subject to measurement errors related to biomechanical properties of the eye such as corneal thickness and curvature.^2^ Topical anesthetics, repeated contact of the tonometer tip against the cornea, eye position, and user performance can also affect IOP readings.^3^^,^^4^ GAT therefore has a variable level of measurement error that limits the accuracy of data, particularly for small and positional IOP changes.^5^ The measurement limitations of GAT are particularly problematic in studying short-term IOP variability and its relevance in glaucoma. IOP is known to vary throughout the day^6^^,^^7^ and also between days.^8^ It is influenced by numerous factors, including physical activity, body position, eye movements, and even gaze direction.^9^^–^^11^

Regarding eye movements, IOP changes have been mainly described in the context of thyroid eye disease.^12^^–^^17^ In both healthy individuals and thyroid eye disease patients, IOP increases during upgaze^11^^–^^13^ and further increases with gaze eccentricity.^11^ The effects of other gaze directions on IOP are less pronounced or contradictory.^12^^,^^13^^,^^18^ In addition, there is only limited knowledge of the effect of eyelid closure on IOP. Eyelid closure elicits an eye movement in many patients (upward and outward), referred to as Bell's phenomenon.^19^ Two studies, performed more than 40 years ago, monitored IOP during eyelid closure and reported a transient small increase in single cases.^20^^,^^21^ Continuous IOP monitoring could further elucidate the dependence of IOP on gaze direction and eyelid closure and clues of underlying mechanisms. To date, however, only a few such studies have been conducted in humans with limited results because of technical limitations of common tonometric methods.^20^^–^^22^ Far more detailed investigations of short-term IOP fluctuations have been conducted in rhesus macaques recently, with the help of an implanted sensor for telemetric recording and readout of IOP. Turner et al.^23^ find that high frequency IOP fluctuations, within the range of milliseconds to seconds, can be mainly attributed to blinking, saccades and the ocular pulse. In nonhuman primates such fluctuations occur throughout the day and can be more than double the baseline IOP, highlighting the dynamic nature of IOP.^23^ The stiffness of the corneoscleral shell of the eye is relevant in the magnitude of short-term IOP fluctuations, and the corresponding fluctuations in ocular strain could be of importance in glaucoma.^24^^–^^26^

The present study applied a similar approach for the first time in humans, investigating primary open-angle glaucoma (POAG) patients who had received an intraocular, telemetric IOP sensor in a previous study.^27^ The sensor allows for continual noncontact IOP recordings independent of user performance and corneal biomechanical properties. Here we present IOP data measured in various gaze directions and during eyelid closure that have not been accessible before in such detail and systematic fashion.

## Methods

### Study Design

The present study is a follow-up to the ARGOS-02 study, which assessed the safety and performance of a novel, telemetric IOP sensor (Eyemate-IO; Implandata Ophthalmic Products GmbH, Hannover, Germany) that was implanted monocularly in the ciliary sulcus at the time of cataract surgery in patients with POAG. The system has since received CE certification and can be used in a clinical setting. A detailed description of the study and validation of IOP readings are given elsewhere.^27^

In brief, the Eyemate-IO system comprises a pressure sensor and a handheld reader device. In the present study, continuous communication between sensor and reader device was established by means of an external antenna attached to the reader and placed around the patient's sensor eye, not touching the eyelids. A figure of the antenna placement around the eye has been published by Al-Nosairy et al.^28^ In this configuration, data acquisition was possible for a maximum of two hours at a sampling rate of approximately 9 Hz.

The current study was conducted at the Department of Ophthalmology of Magdeburg University Hospitals. The study protocol adhered to the tenets of Helsinki and was conducted with local ethics committee approval. Patients provided written informed consent after a detailed explanation of the study prior to participation.

### Participants

Participants were a subset of 22 POAG patients implanted with the intraocular pressure sensor during the ARGOS-02 study at least 3 years ago, with relatively strict inclusion and exclusion criteria.^27^ Eleven patients were willing and able to take part in the present study.

Inclusion criteria:

1. Mentally competent and willing to provide written informed consent
2. Male or female aged 50 to 85 years
3. Functional Eyemate-IO sensor

Exclusion criteria:

1. Nonfunctioning Eyemate-IO sensor
2. Severe general diseases that make the participation in most of the examinations impossible in the investigator's opinion
3. Ocular diseases, which preclude the comparative measurements of the IOP (e.g., corneal ulcer, corneal scar, keratoconus, severe irregular astigmatism)
4. Paralysis of the outer ocular muscles, which preclude the IOP measurements in different viewing directions

All 11 patients (age 61-78; five female), who were diagnosed with POAG for up to 34 years, were eligible and thus included in the present study. The Eyemate-IO system was functional in all patients and calibrated to within 2 mm Hg of GAT measurements. Data on demographics and other patient details are given in Table 1^2^. There were no other eye diseases apart from POAG, nor oculomotor disorders such as strabismus or active thyroid disease in the study group.

**Table 1.** Patient Characteristics

Characteristic	Study Eye (n = 11)	Fellow Eye (n = 11)
Age [years]	72 ± 5	
Gender		
Male	6 (55)	
Female	5 (45)	
Glaucoma stage^*^		
Early	8 (73)	11 (100)
Moderate	2 (18)	—
Severe	1 (9)	—
Sensor in the right eye	8 (73)	3 (27)
BCVA (ETDRS letter score)	83 [79, 86]	82 ± 4
MD [dB]	−3 [−6, −1]	−2 ± 2
VFI [dB]	95 [90, 99]	97 [92,99]
PSD [dB]	2.5 [1.5-6.5]	2.0 [1.7-3.9]
CCT [µm]	568 ± 49	566 ± 44
Glaucoma surgeries^†^		
0	9 (82)	9 (82)
1	1 (9)	2 (18)
2	1 (9)	—
Glaucoma medications		
0	4 (36)	3 (27)
1	1 (9)	2 (18)
2	3 (27)	3 (27)
3	3 (27)	3 (27)

Normal distributed continuous data presented as mean ± SD, non-normal distributed data presented as median [Interquartile range] and counts presented as number (%).

BCVA, best-corrected visual acuity (using ETDRS letters); MD, mean defect in visual field, VFI, Visual field Index (Humphrey Field Analyzer III); PSD, Visual field–Pattern Standard Deviation; CCT, Central Corneal Thickness.

* One female patient of 77 years was staged with severe glaucoma in the study eye (BCVA= 58, MD=-21.46 and PSD = 13.23 dB, left eye). Two patients were staged with moderate glaucoma in the study eye, one male of 78 years (BCVA = 88, MD= -10.29, PSD =8.81, left eye) and one female of 76 years (BCVA = 86, MD = −6.98, PSD = 6.4, right eye). The other 8 patients were staged with mild glaucoma in the study eye.

† One patient had previously undergone selective laser treatment in the study eye. Another patient had trabeculectomy and selective laser trabeculoplasty in the study eye. The filtering bleb in the latter patient was not functional (being flat and scarred), and pressure-lowering medication was necessary at the time.

**Table 2.** IOP-Comparison for All Gaze Positions

	10^*^ IOP [mm Hg]^†^				20^*^ IOP [mm Hg]^†^				25^*^ IOP [mm Hg]^†^			
Gaze	Position	Baseline	Difference	*P* Value^‡^	Position	Baseline	Difference	*P* Value^‡^	Position	Baseline	Difference	*P* Value^‡^
S	18.6 ± 1.26	17.5 ± 1.47	1.1 ± 0.61	0.110	20.8 ± 1.47	16.9 ± 1.35	3.9 ± 1.38	0.018	21.4 ± 1.53	16.9 ± 1.25	4.4 ± 1.48	0.014
ST	17.6 ± 1.33	16.7 ± 1.44	0.87 ± 0.28	0.011	19.2 ± 1.30	16.6 ± 1.30	2.6 ± 0.70	0.004 (0.008)	20.1 ± 1.32	17.1 ± 1.25	2.9 ± 0.94	0.011
T	17.3 ± 1.39	16.7 ± 1.41	0.60 ± 0.083	<0.001 (0.006)	17.6 ± 1.39	16.5 ± 1.43	1.1 ± 0.36	0.012	18.1 ± 1.29	16.9 ± 1.32	1.2 ± 0.27	0.001 (0.007)
IT	16.9 ± 1.45	16.5 ± 1.45	0.33 ± 0.14	0.039	16.8 ± 1.44	16.3 ± 1.44	0.47 ± 0.12	0.003 (0.007)	16.9 ± 1.34	16.7 ± 1.31	0.14 ± 0.27	0.603
I	16.3 ± 1.56	16.6 ± 1.44	−0.31 ± 0.17	0.098	15.8 ± 1.50	16.3 ± 1.45	−0.45 ± 0.23	0.074	15.7 ± 1.43	16.3 ± 1.35	−0.55 ± 0.31	0.110
IN	15.8 ± 1.45	16.5 ± 1.44	−0.71 ± 0.16	0.001 (0.007)	15.1 ± 1.46	16.2 ± 1.40	−1.1 ± 0.18	<0.001 (0.006)	14.9 ± 1.36	16.5 ± 1.35	−1.6 ± 0.23	<0.001 (0.006)
N	16.2 ± 1.42	16.4 ± 1.44	−0.18 ± 0.17	0.309	15.5 ± 1.40	16.2 ± 1.40	−0.71 ± 0.47	0.161	15.8 ± 1.44	16.4 ± 1.29	−0.51 ± 0.67	0.420
SN	17.1 ± 1.36	16.4 ± 1.42	0.66 ± 0.42	0.143	17.9 ± 1.50	16.0 ± 1.39	1.9 ± 0.88	0.061	18.9 ± 1.63	16.2 ± 1.20	2.9 ± 1.40	0.083
Overall^§^	17.0 ± 0.48	16.7 ± 0.49	0.29 ± 0.12		17.3 ± 0.52	16.4 ± 0.47	0.97 ± 0.29		17.7 ± 0.53	16.6 ± 0.44	1.1 ± 0.36	

Normal distributed continuous data presented as mean ± SEM.

S, superior; ST, superior temporal; T, temporal; IT, inferior temporal; I; inferior; IN, inferior nasal; N, nasal; SN, superior nasal.

^*^Acquired IOP data during gaze experiments presented for each eccentricity separately (N = 11, mean over 3 repetitions).

† IOP values shown (from left to right) for each gaze position, preceding primary gaze as baseline measurement, and the difference between each position and preceding baseline (ΔIOP).

‡ A Bonferroni-Holm correction was applied for eight paired *t*-tests to reduce the alpha error and is depicted in brackets.

§ IOP values and differences (ΔIOP) were averaged over all gaze positions and baselines for each eccentricity separately (Overall row).

### Procedures

All patients underwent a comprehensive ophthalmological examination before study procedures, including best-corrected visual acuity (BCVA) by ETDRS letter charts, visual fields (Humphrey Field Analyzer III), corneal pachymetry, slit-lamp biomicroscopy and fundoscopy. Glaucoma was staged according to the Hodapp classification.^29^

### Experimental Procedure

**Figure 1.**
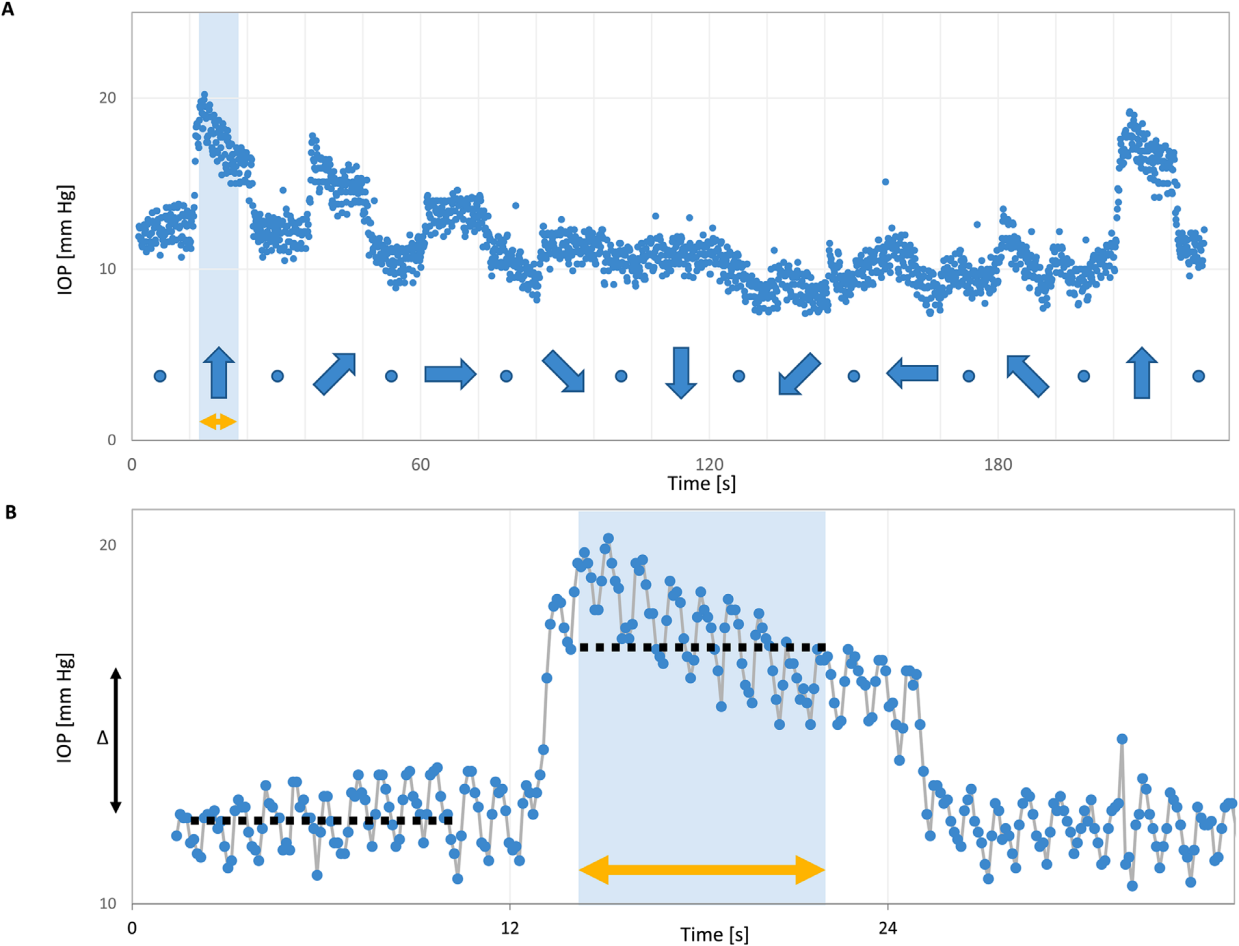
Example IOP recording and analysis during a gaze experiment. **(A**) IOP recording of one patient while alternating eccentric gaze positions with primary gazes at 25°. The scatter of data points in each position of about 2 mm Hg comprised IOP changes that coincided with heart-rate and are therefore related to the ocular pulse amplitude rather than noise. **(B)** Depiction of ocular pulse and IOP analysis. The ocular pulse is visible within IOP variability that occurs within several seconds (*gray line*). Change in mean IOP was calculated by subtracting mean IOP during each gaze direction from primary gaze. Hence, all IOP measurements refer back to the baseline, acting as an internal reference to reduce noise from various sources of variability on measurements. Negative and positive ΔIOP denote an IOP decrease and increase, respectively, for eccentric gaze compared to the primary position. In A, blue arrows indicate gaze directions and blue dots indicate primary gazes, all in 12 s intervals. In both A and B, yellow arrows indicate the 8 s analysis intervals. The two time windows ‘TW_initial_’ and ‘TW_final_’ used for time course analysis of IOP included the initial and final 2 seconds of each eccentric gaze position analysis interval.

Patients were seated in front of a Harms wall (Heuser, model Diagonal Blue, 2.35 m × 2.35 m with a 25° model) at a distance of 2.5 m in a standard chin rest with a head strap and the center of the wall (primary position) at eye height. One investigator instructed the patients with regard to gaze directions, while a second person in the room video recorded the experiment and instructed patients to maintain a stable head position throughout the experiment. After an initial baseline recording in primary position patients were directed to fixate on eight positions at three eccentricities (10°, 20°, and 25°, respectively) on the Harms wall for 12 seconds each (Fig. 1A), always starting with an upward gaze at a specific eccentricity and then proceeding clockwise through each position for that eccentricity. Between each position, the patients returned their gaze to the primary position for 12 seconds as a baseline measurement. The superior gaze and primary position were repeated at the end of each experiment as an internal control. Experiments consisted of three repetitions to minimize artefacts from blinking, breathing, and small body movements.

### Missing and Excluded Measurements

In two patients, superonasal gaze positions at 25° placed the sensor out of alignment with the antenna and led to a reduced number of IOP measurements in two out of three repetitions (Supplementary Fig. S1, patients 6 and 8). One of those patients also had missing measurements in the nasal gaze position at 25° in one of three repetitions. In one patient (patient 5), only two repetitions for each experiment could be performed. Coughing led to the exclusion of one repetition at 20° in one patient (patient 6) and the exclusion of one repeated upward gaze at 25° in another patient (Supplementary Fig. S1, patient 4).

### Assessment of Eyelid Closure

After the initial experiments, data were acquired on IOP response on eyelid closure to explore the effect of the eyelid on IOP. A subset of nine patients was instructed to close their eyes for 10 seconds for four times after baseline measurement.

### Data Analysis and Statistics

Because the primary focus of our investigation was the IOP during fixed gaze positions, the first and last two seconds of each gaze position were excluded from analysis. This was done to avoid including IOP transients during eye movements, which—because of differences in reaction time of the patients—were not always precisely aligned with the 12-second time interval allotted for each gaze position. Representative IOP values at the eight different gaze positions for each eccentricity were obtained by (1) calculating the mean IOP for each position (eight-second interval) and (2) determining the IOP response (ΔIOP) by subtracting the mean IOP of the preceding baseline at the primary position (Fig. 1B). The Shapiro Wilk test did not reject the null hypothesis for normally distributed data and Q-Q plots confirmed normality. A χ^2^ test was used to test the independence between group means. A three-way factorial repeated-measures ANOVA was performed with ΔIOP as a dependent variable and gaze direction, eccentricity, and repetition of the experiment as fixed variables. For the majority of data that did not meet the assumption of sphericity (tested using Mauchly's test), a Greenhouse-Geisser correction was applied. Post-hoc *t*-tests, Bonferroni-Holm corrected^30^ for multiple testing, were applied to assess the statistical significance of the ΔIOPs for each gaze position. In addition, a paired *t*-test was performed to compare the first and last upgaze of each experiment as an internal control.

### Exploratory Individual Analysis

Because of an observed large interindividual variability in the magnitude of gaze-dependent IOP changes, an exploratory analysis was performed to investigate whether the direction of IOP change was similar among patients. Because the study was optimized for group-level analyses and consequently underpowered for an individualized analysis (e.g., three repetitions per condition), we reduced the parameter range: at the group level we identified the two gaze directions with the greatest IOP increase and decrease, respectively, and compared them for each individual with a paired *t*-test. ANOVA on the raw time series data was not applicable, because the homoscedasticity requirement was violated.

### Time-Course of IOP Within Epochs of Stable Gaze Positions

The IOP change during each gaze position was found to drift after an initial saccadal spike in most patients. We performed an additional analysis to explore the time course of the IOP change for each gaze position and calculated the difference in mean IOP of a smaller time window at the beginning and end of each epoch. The two new time windows included the initial and final two seconds of each eccentric gaze position's analysis interval. Average ∆IOP for the beginning (initial Time Window or TW_initial_) and end (final Time Window or TW_final_) of each gaze position, as well as the difference (d∆IOP) between the two values, are given for the average across patients in Supplementary Figure S3. In addition, in Supplementary Figure S4 the correlation between TW_initial_ and d∆IOP is depicted.

### Comparison of IOP Dependence on Eyelid Closure and Gaze

**Figure 2.**
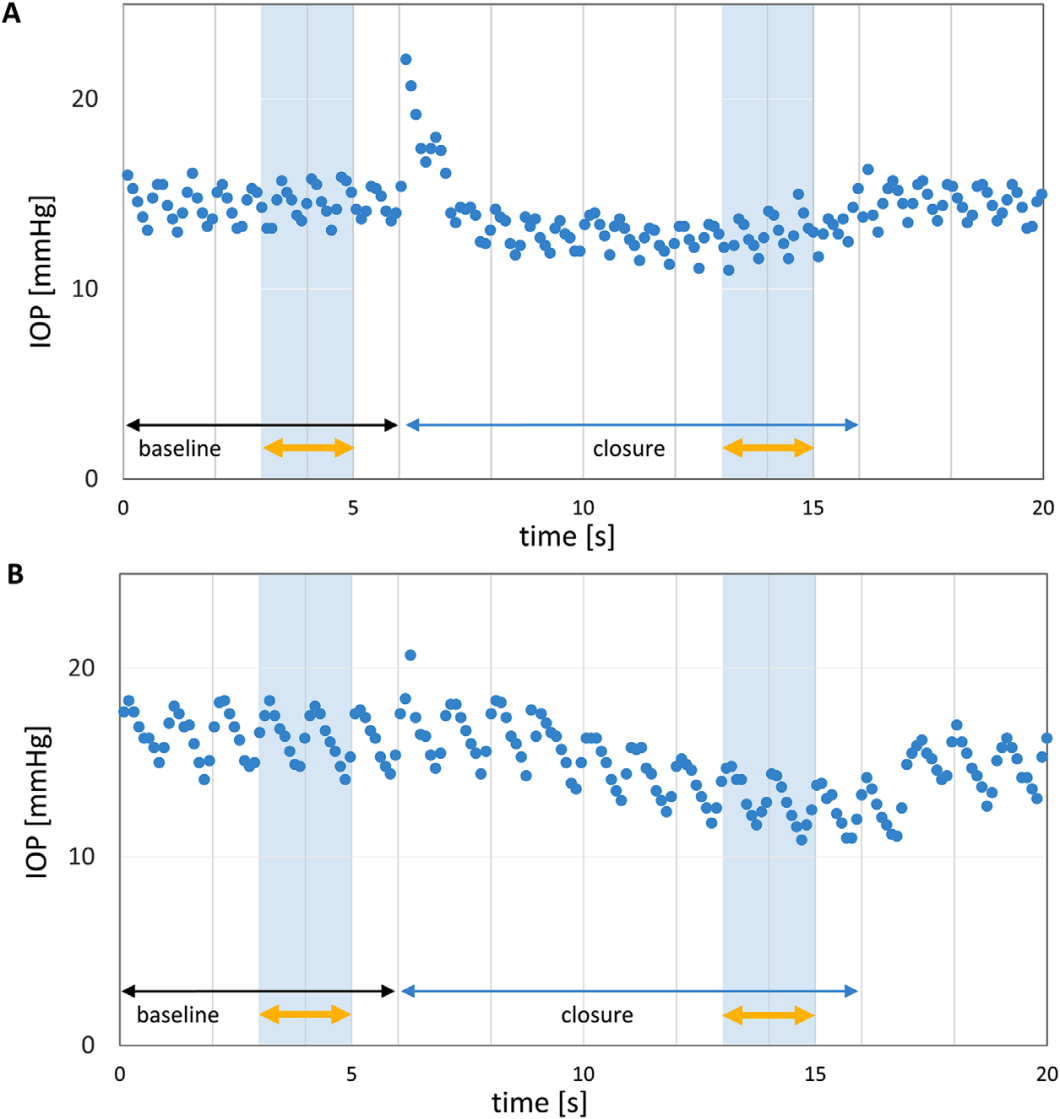
IOP time courses for two representative patients upon eyelid closure. **(A)** Patient with IOP drop immediately after an initial IOP peak. **(B)** Patient with delayed IOP drop after a moderate initial increase. Yellow errors indicate the analysis intervals for the quantitative assessment of eyelid closure depicted in Figure 4.

For better comparability across conditions only the last two seconds of each epoch of eyelid closure and gaze were included. As seen in Figure 2, eyelid closure led to an initial IOP increase in most patients. Similar IOP spikes could be seen during deliberate blinking, but not during incident (involuntary) blinking (data not shown). Because this might be an indication for additional tension of the eyelid muscles for the deliberate act of closing the eyelids, we chose to exclude this initial spike and focused on the last part of the closure interval, when the muscles had presumably relaxed into a steady state similar to that during sleep. A paired *t*-test was performed to compare ΔIOP between primary gaze position and closed eyelids. For the same subset of patients, ΔIOP for superonasal, superior and superotemporal gaze was averaged to represent gaze “upward.” The ΔIOP for inferotemporal, inferior, and inferonasal gaze were averaged to present gaze “downward” for each eccentricity. These ΔIOPs were determined for the three eccentricities, that is from 25° superior to 25° inferior (Fig. 4) and compared via a repeated-measures ANOVA with ΔIOP as a dependent variable and gaze direction and eccentricity as fixed variables. Finally, ΔIOP at 25° inferior was compared with ΔIOP during eyelid closure using a paired *t*-test. *P* ≤ 0.05 was considered statistically significant. Analyses were performed in Matlab 2020b (The Mathworks Inc., Natick, MA, USA) and included the use of the Superbar file exchange and the Gramm Toolbox.^31^^,^^32^

## Results

### Effect of Gaze Direction on IOP

A typical IOP recording is depicted in Figure 1. It demonstrates systematic IOP changes of the high temporal resolution recordings, including those associated with the ocular pulse as indicated in Figure 1B. IOP changes induced by upgaze typically comprised an initial IOP spike during each saccadal movement toward a new gaze direction, followed by a slow decline approaching a plateau phase. After returning to primary gaze, IOP returned to baseline levels. Downgaze induced an IOP decrease. Although these general features were largely similar across patients, the magnitude of IOP responses varied among patients, as shown in the first run of all gaze-related IOP recordings in Supplementary Figure S1.

**Figure 3.**
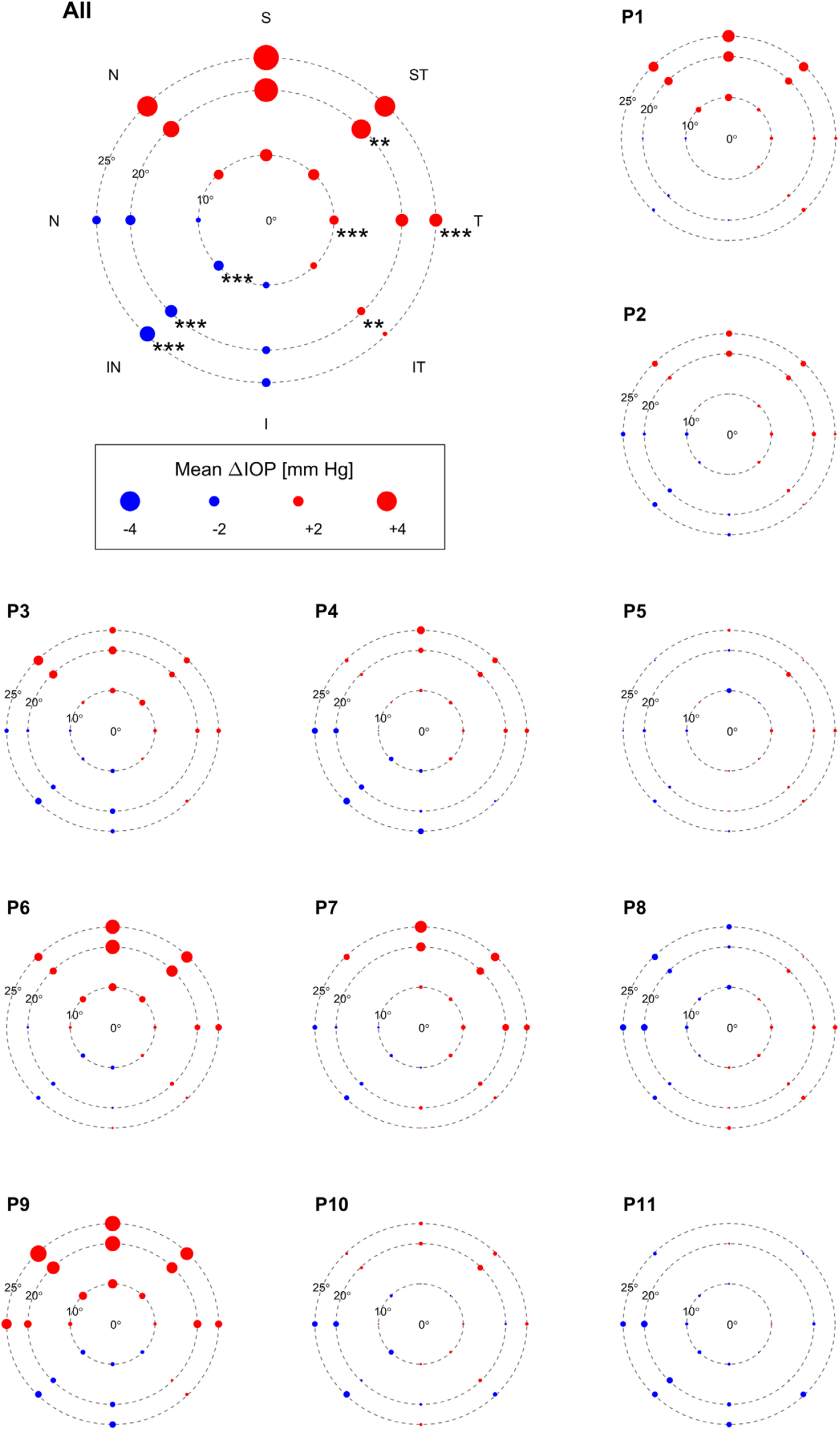
Mean Δ IOP (n=11) for each gaze direction and eccentricity. Mean ΔIOP for each gaze direction and eccentricity at the group level (top left) and for each individual separately. Gaze directions in eight directions are presented as ‘S’ (Superior), ‘ST’ (Superior Temporal), ‘T’ (Temporal), ‘IT’ (Inferior Temporal), ‘I’ (Inferior). ‘IN’ (Inferior Nasal), ‘N’ (Nasal) and ‘SN’ (Superior Nasal). Magnitude of IOP-change scales with disc diameter. The discs in the individual plots are scaled down linearly from the size key with the size of the plots. For the group level statistics, asterisks indicate significance of paired T-tests corrected for multiple tests (*P*≤ .01 = **, *P*≤ .001 = ***). Marked IOP increase for upward gazes is visible especially for patients 1, 6, 7 and 9. Overall, most patients show similar trends of IOP increase or decrease in similar directions and eccentricities, for a detailed assessment see Supplemental Table S1.

The gaze-related IOP changes were quantified by calculating the IOP-difference between the specific gaze direction and the previous primary position, termed ΔIOP (Table 2, see Methods). A repeated-measures ANOVA (fixed factors gaze direction, eccentricity, experiment repetition) demonstrated a significant effect of gaze direction (*P* = 0.011) and experiment repetition (*P* = 0.004), with lower ΔIOP for the second recordings (mean ± SEM [mm Hg]: 0.571 ± 0.158 vs. 0.898 ± 0.170 and 0.916 ± 0.177). There was a significant interaction between gaze direction and eccentricity (*P* = 0.003) as evident from Figure 3 and Supplementary Figure S2. IOP strongly depended on gaze direction on the group level with the highest mean increase occurring at 25° upgaze (ΔIOP mean ± SEM [mm Hg] = 4.2 ± 1.48) and the largest mean decrease at 25° inferonasal gaze (ΔIOP = −1.63 ± 0.23). Post-hoc *t*-tests (see Fig. 3 and Table 1) revealed that the IOP decrease in inferonasal gaze was statistically significant at all eccentricities (10°: *P* = 0.001, 20° and 25°: *P* < .001), whereas the IOP increase was significant only for 20° superotemporal gaze (*P* = 0.004), but not for upward (superior) gaze at any eccentricity. Remarkably, the largest mean IOP increase was not statistically significant. This is likely associated with the large interindividual differences in ΔIOP for upgaze as evident from Figure 3 and Supplementary Figure S1. Although the largest individual ΔIOP observed at 25° superior gaze was 13.1 mm Hg, three patients (patients 5, 10, and 11) had a ΔIOP of less than 1 mm Hg for this condition, one (patient 8) even had a negative ΔIOP (−1.6 mm Hg). An exploratory analysis comparing these two gaze positions (inferonasal and superior) at the individual level as detailed in Methods showed a significant difference in these positions in eight of 11 individuals, with one further patient barely missing statistical significance. ΔIOPs and *P* values are shown in Supplementary Table S1. No significant correlations of the individual gaze dependent ΔIOP at 25° with visual field parameters (Mean Deviation (MD): *R*^2^ = 0.01, *P* = 0.77, Pattern Standard Deviation (PSD): *R*^2^ = 0.03, *P* = 0.60) or baseline IOP (*R*^2^ = 0.15, *P* = 0.22) were observed.

Each experimental series started and ended with a superior gaze, which consequently served as a control to assess sequential effects. Sequential effects on ΔIOP were absent for all eccentricities (mean ± SEM [mm Hg] at 10°, 20°, and 25°: −0.11 ± 0.21; *P* = 0.62; 1.0 ± 0.49, *P* = 0.059; 25°: 0.23 ± 0.39, *P* = 0.57; paired *t*-tests).

To further explore the tendency of IOP to decrease after an initial spike when taking a new gaze position, as depicted in Figures 1 and 2, we calculated ΔIOP for the first and last two seconds (TW_initial_ and TW_final_ respectively) within each analyzed gaze position interval. There was a drop in IOP between TW_initial_ and TW_final_ in all but one gaze position (IT), as visualized in the difference “dΔIOP” (Supplementary Figure S3). This decrease in dΔIOP was not only observed after an initial increase in the upward and temporal gaze positions, but also during the downward and nasal gaze positions, thus constituting an increase in deviation from baseline over time in these latter positions. Overall, we found a significant correlation between TW_initial_ and dΔIOP, that is the higher the initial change in IOP the larger the subsequent drop while holding the gaze steady (*R*² = 0.56, *P* < 0.01, Supplementary Figure S4).

### Effect of Eyelid Position on IOP

**Figure 4.**
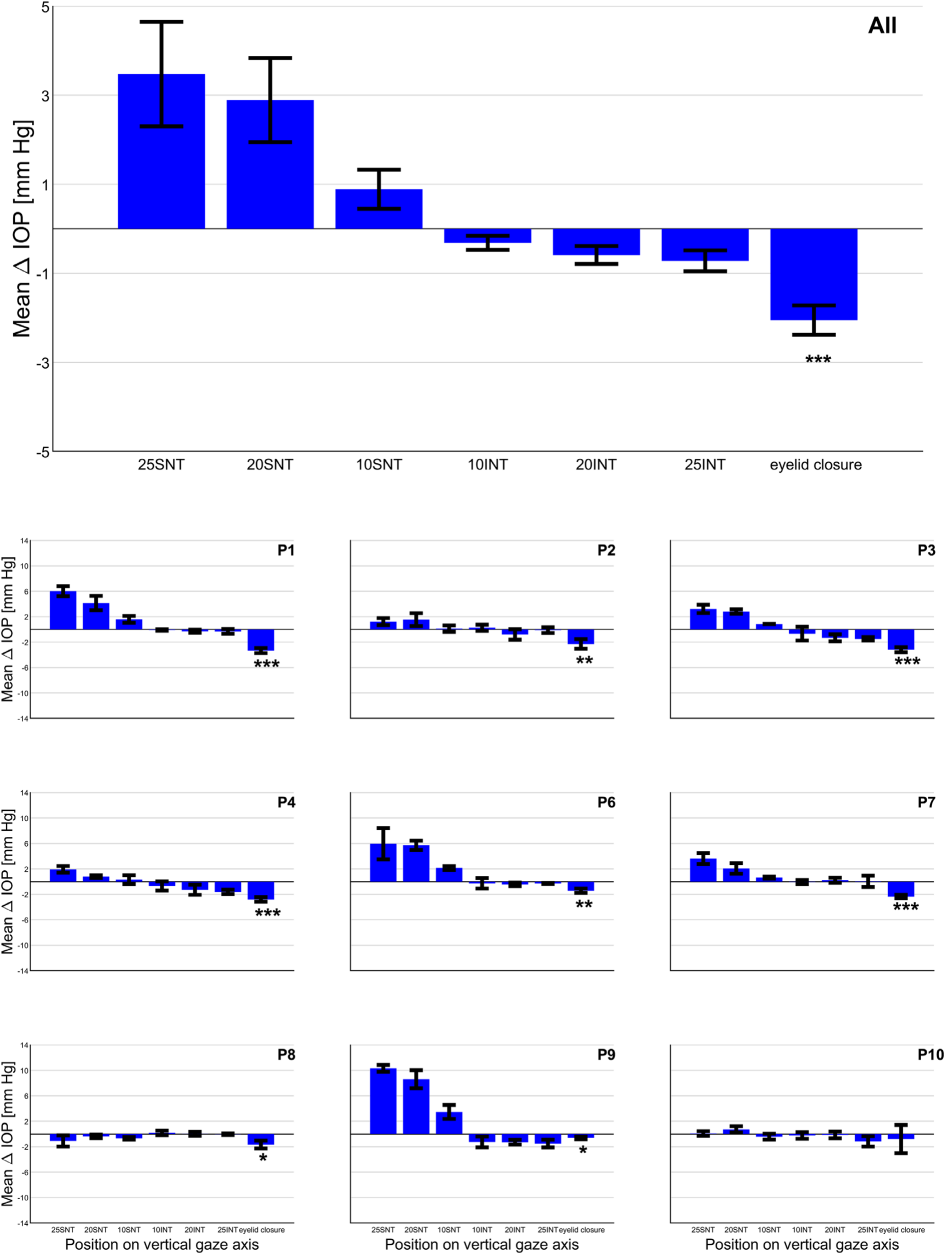
Comparison of mean ΔIOP in vertical gaze directions with eyelid closure. Mean ΔIOP ± SEM for upgaze (‘SNT’, i.e. superior nasal to superior temporal: average ΔIOP across directions SN, S and ST) at the three eccentricities, followed by downgazes or ‘INT’ (superior nasal to superior temporal), and lastly for eyelid closure. As a result, from left to right the position of the initially extracted eyelid moves further down for each condition. Data on gaze (n=11) and eyelid closure (n=9) are averages of 3 and 4 repetitions, respectively. For details on the analysis see Methods. One-sample T-tests were performed for each mean ΔIOP to test the difference to “0” on the group level. *P*-values (Bonferroni-Holm adjusted alpha level) from left to right: 25SNT: *P* = 0.02 (0.02), 20SNT: *P*= 0.02 (0.01), 10SNT: *P* = 0.08 (0.03), 10INT: *P* = 0.08 (0.05), 20INT: *P* = 0.02 (0.01), 25INT: *P* = 0.02 (0.008), eyelid closure: *P* <0.001 (0.007). Hence, the only significant P-value after correction was for eyelid closure and is indicated in the top figure. In addition, paired T-tests compared each gaze position on the vertical axis with eyelid closure (not shown). P-values (Bonferroni-Holm adjusted alpha level) from left to right: 25SNT, *P* = 0.002 (0.01), 20SNT *P*<0.001 (0.01), 10SNT *P*<0.001 (0.008), 10INT *P*=0.002 (0.02), 20INT *P*= 0.004 (0.03), 25INT *P*=0.01 (0.05). Individual plots (Mean ΔIOP ± SD) depict a similar trend in decreasing ΔIOP from upward to downward gaze and eyelid closure in most patients and the significances for eyelid closure vs. baseline as determined with paired t-tests (*P*< .05 = *, *P*≤ .01 = **, *P*≤ .001 = ***), as detailed in Supplemental Table S2.

Investigating the effect of eyelid closure on IOP in 9 patients, we found a significant IOP decrease during eyelid closure (mean ± SEM [mm Hg] : −2.1 ± 0.33; *P* < 0.001) compared to primary gaze. Individual decreases ranged from 0.6 to 3.3 mm Hg (Supplementary Table S2). Subsequently, we performed an equivalent analysis of the gaze data in the subset of nine patients with eyelid closure data, that is, with comparable two-second intervals at the end of each gaze period (see Methods). The IOP for superonasal, superior, and superotemporal gaze were averaged into one mean IOP for upgaze (termed “SNT”) for each eccentricity, and accordingly for downgaze (termed “INT”) as shown in Figure 4 (depicted as group and individual data). SNT-ΔIOPs exceeded ITN-ΔIOPs in an eccentricity-dependent manner (repeated measures ANOVA [fixed factors *gaze direction* and *eccentricity*]: significant factors *gaze direction* [*P* = 0.015] and *eccentricity* [*P* = 0.03], significant interaction of *gaze direction* and *eccentricity* [*P* = 0.002]). Importantly, a paired *t*-test comparing the effect of eyelid closure and downgaze at 25° (mean IOP ± SEM [mm Hg]: −2.1 ± 0.33 and −0.72 ± 0.24, respectively) revealed a greater IOP decrease for eyelid closure (*P* = 0.014).

## Discussion

Using continual telemetric IOP monitoring, this study provides the first systematic quantitative assessment of IOP changes associated with changes in gaze direction and with eyelid closure in POAG patients. Our data show IOP to respond highly dynamically to changes in gaze direction with ocular movement causing initial spikes in IOP that trail off plateau-like while holding a gaze position. For better comparison with previous studies we chose to focus on the average IOP during the fixed gaze positions, excluding the brief peak IOPs during eye movement from analysis. Using this approach, we observed that upward and temporal gaze positions induced an IOP increase for all tested eccentricities, whereas downward and nasal gaze positions induced an IOP decrease, suggesting an asymmetrical gaze position−dependent IOP response. This pattern was prominent in eight of the 11 individuals tested, with large variability between patients.

During eyelid closure, IOP decreased by 2.1 mm Hg from primary gaze position, with individual decreases ranging from 0.6 to 3.3 mm Hg. When looking at the combined gaze experiment data from upward to downward gaze and then eyelid closure, a progression of ΔIOP from increase to decrease emerges, which suggests that eyelid pressure on the eye globe may also contribute to IOP. Overall, our observations considerably expand previous reports on the interaction of gaze or eyelid closure on IOP in human eyes.^11^^–^^18^^,^^20^^,^^21^ It must be noted that the previous investigations had discrepant outcomes and were heterogeneous in their approaches. The difference in measuring methods may be the main source of some discrepancies between our results and those previously reported.

### Impact of Horizontal Gaze Directions on IOP

We found an IOP increase during temporal gaze, as observed by others,^18^^,^^21^ and a decrease in nasal gaze that largely deviates from previous studies.^12^^,^^18^^,^^20^^,^^21^ Concerning the latter, Moses et al.^18^ measured with GAT on the central cornea at up to 50° of eccentricity in six healthy college students (no exact age given). In the present study, we did not measure at such large deviations from the primary gaze, and glaucoma patients with an average age of 72 years were included. More qualitative in nature were the measurements of Cooper et al.,^21^ who used an applanation pressure transducer placed in the lower fornix of a healthy volunteer, and Coleman et al.,^20^ who placed a needle probe transducer in the anterior chamber of an eye before enucleation resulting from a tumor. Therefore the source of this discrepancy is not currently discernible. However, an asymmetric effect of horizontal gaze is to be expected considering that the active force of the medial rectus muscle is 40% stronger compared to the lateral rectus.^33^ In line, Moses et al.^18^ also found a smaller IOP increase during nasal gaze than during temporal gaze. Further investigations are warranted to explain the observed IOP decrease during nasal gaze in our patient group.

### Impact of Vertical Gaze on IOP

The eccentricity-dependent increase in IOP with upgaze in the present study has previously been reported for healthy subjects, as well as for patients with either glaucoma or autoimmune thyroid disorders.^11^^–^^15^ Although Herzog et al.^14^ dispute the accuracy of GAT-measurements during upgaze, because of the tonometry prism being placed on the peripheral cornea or even sclera, our measurements were largely unaffected by these constraints and corroborate the expected, albeit interindividual variable, increase in IOP. Such variability has also been previously reported.^11^^–^^15^ Notably, one patient in our data set even showed a reproducible decrease in IOP during upgaze.

We observed an eccentricity-dependent decrease in IOP in downgaze, which was also observed by Zappia et al.^13^ and Nardi et al.^12^ with similar values and at similar eccentricities (−0.34 mm Hg at 15° and −0.52 mm Hg at 22°, respectively). Whitacre and Stein^5^ described the decrease as a tonographic effect, because Zappia and Nardi always measured upgaze first. In the present study, however, we support the observed IOP decreases excluding a possible tonographic effect, because returning to upgaze at the end of each experimental sequence showed no difference to the first upgaze. In contrast, Reader et al.^11^ observed a minimum IOP in superior gaze at −6.7° and an IOP increase for downgaze (1.5 mm Hg at 20°), whereas our data show a further IOP decrease. The reason for this difference is likely related to Herzog's findings^14^ that measurements on the corneal periphery are probably affected by greater corneal thickness and rigidity, leading to false-high readings.

### Time-Course of IOP Within Epochs of Stable Gaze Positions

Throughout the experiment, IOP showed the largest and fastest changes during ocular movement and had the tendency to slowly decrease thereafter while the gaze was held steady. We observed that the first two seconds of each epoch had higher IOP readings compared to the last two seconds. The IOP increase in upward gaze was significantly higher compared to baseline in the first time window, whereas it was not significant in the last two seconds or in the entire eight-second analysis period. In addition, the decrease of IOP over time within each gaze period was significantly correlated with the initial IOP response that occurred on a change in gaze direction. The dynamic IOP response to perturbation has been observed before^20^^,^^23^^,^^26^ and probably reflects a pressure-dependent increase in aqueous outflow when IOP is increased. Whether this IOP response is purely passive or due to regulatory changes in the outflow system is part of an ongoing debate.^34^ A model-driven analysis approach or defined experimental IOP manipulation may potentially yield more information about the outflow system in future studies with the telemetric IOP sensor.

Because our main focus was on IOP during steady gaze positions rather than on transient IOP spikes during ocular movement, we did not quantify the initial peak IOP on change of gaze direction or eyelid closure. This decision was partly due to the fact that although the 9 Hz sampling rate of the sensor is sufficient to capture the ocular pulse amplitude as seen in Figures 1 and 2, it may be too low to fully capture the true IOP spike during saccades of the eye, depending on timing of the ocular movement. Future studies with more frequent saccades (e.g., while reading or watching video sequences on a large screen), ideally in combination with an eye tracker, may better elucidate the effects of ocular movement than the current study. A higher sampling rate like the 500 Hz of the device used by Downs et al.^24^^,^^26^ likely also has a better chance of fully capturing minute IOP transients.

### Impact of Eyelid Closure on IOP

The pull of the extraocular muscles is likely a major contributor to the observed IOP responses. However, the anatomical asymmetry between the superior and inferior rectus muscle is relatively small,^35^ implying that the effect of their alternate activation/relaxation should be similar in vertical gaze, that is, an increase both during upward and downward gaze. We, however, observed a decrease of IOP for downgaze, which motivated us to explore the hypothesis that eyelid position may contribute to the IOP differences seen.

Moses et al.^36^ had previously measured an increase in IOP on voluntary wide opening of the eyes and speculated that the lid retraction by the *musculus levator palpebrae superioris* would cause the lid tissue to press on the eye. A similar lid retraction occurs during upgaze, whereas during downgaze, the lid relaxes and spreads over the eye globe without tension, leading to the eccentricity-dependent IOP decrease that we observed. In line, closing the eyelids completely led to an even greater drop in IOP in the present study.

In contrast, both Cooper et al.^21^ and Coleman and Trockel^20^ reported large increases in IOP on eyelid closure. By means of a transensor that applanated the inferior sclera underneath the eyelid, Cooper et al.^21^ managed to apply indirect tonometry while the eyes were closed. An IOP increase by approximately 7 mm Hg was observed while ignoring blink artifacts before the maneuver. The data of Coleman and Trockel^20^ involved recordings intraocularly and depicted an IOP trend that increased by about 6 mm Hg over five seconds without an initial prominent IOP spike. The force applied to close the eyes may, in part, be underlying the observed discrepancy.^37^^,^^38^ The presented study minimized a possible effect of forceful eyelid closure on IOP by both patient instruction and data analysis. In addition, IOP is likely also affected by Bell's phenomenon.^19^ This upward and outward movement of the globe during eyelid closure is similar to superotemporal gaze, which led to an IOP increase in the present study. However, the expected IOP increase did not appear to counteract the proposed IOP decreasing effect of eyelid closure. This would support the idea that Bell's phenomenon may reflect a release of muscle tension, thus minimizing the effect of extraocular muscle pull on IOP.^39^^–^^41^ Because our research did not explicitly investigate the role of Bell's phenomenon and its presence or absence in our study patients, more research is needed specifically designed to investigate the interplay of eyelid position, Bell's phenomenon, and extraocular muscle pull on IOP.

### Clinical Relevance of Gaze and Eyelid Position Dependence of IOP Measurements

Here we demonstrate that IOP is sensitive to ocular movements and the direction of gaze in glaucoma patients. IOP readings should therefore be performed under similar eye positions if possible to reduce gaze-related variability. Large effects could be seen in some individuals, whereas others showed only limited responses to different gaze positions. We tried to explore whether the magnitude of IOP response potentially affects glaucoma susceptibility, as larger IOP variability is thought to be a relevant independent risk factor for glaucoma progression.^42^ Although we did not find a statistically significant correlation between the IOP response and the mean defect in the visual fields, this lack of association may very well be a result of the small number of patients in our study. Both cross-sectional and longitudinal studies with much larger study populations will be needed to further investigate the pathophysiological relevance of gaze related IOP response. Interestingly, the same patient group demonstrated a more homogenous IOP response to changes in body position in another study.^28^ It is possible that IOP responses during ocular movements are more subject-dependent than for other influencing factors such as body posture. Because more patients will likely be implanted with the Eyemate, it will be easier to investigate short-term IOP variability in response to various external stimuli, as well as physiological parameters in future studies. Currently, however, investigations are limited to a small and heterogeneous group of glaucoma patients.

## Conclusion

We demonstrate a potential dependency of IOP on gaze direction in glaucoma patients using telemetric IOP monitoring, with the highest readings in upgaze and lowest readings during inferonasal gaze and eyelid closure, albeit with large interindividual variability, the clinical significance of which remains to be explored. We also found an unexpected asymmetry between nasal and temporal gaze. Our data suggest a complex interplay of extraocular muscles with other orbital structures such as the eyelid. The observed dynamic and individual IOP responses on changes in gaze direction motivate further study of underlying biomechanical and glaucoma-related mechanisms.

## Supplementary Material

Supplement 1

Supplement 2

Supplement 3

Supplement 4

Supplement 5

Supplement 6
