# Supplementary material for: Implanted Microsensor Continuous IOP Telemetry Suggests Gaze and Eyelid Closure Effects on IOP—A Preliminary Study
Source: Invest Ophthalmol Vis Sci. 2021 May 6;62(6):8. doi: 10.1167/iovs.62.6.8 (PMC8107486; doi:10.1167/iovs.62.6.8)
Supplement: Supplement 1 [file iovs-62-6-8_s001.pdf]

first run of continuous IOP recordings

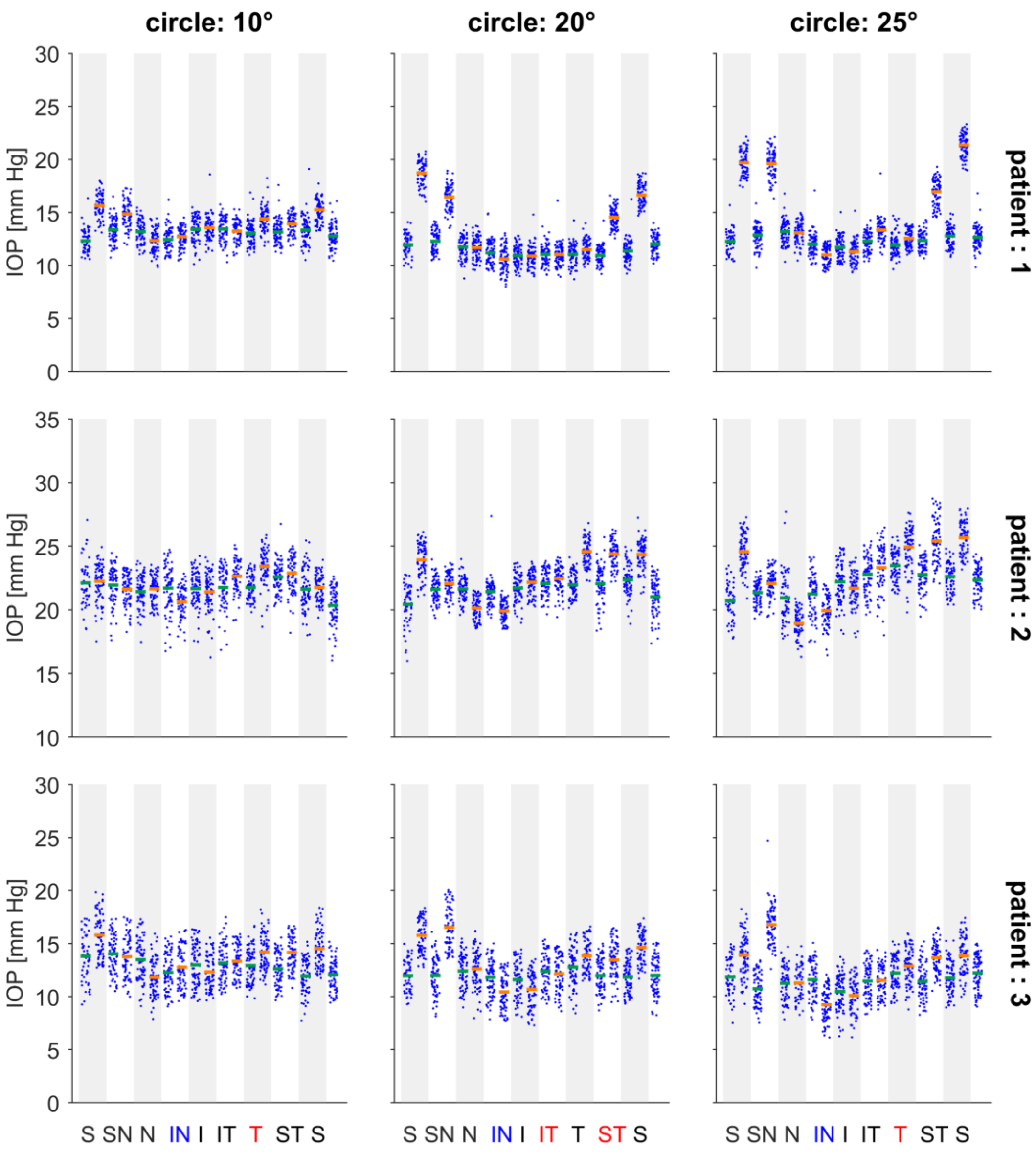

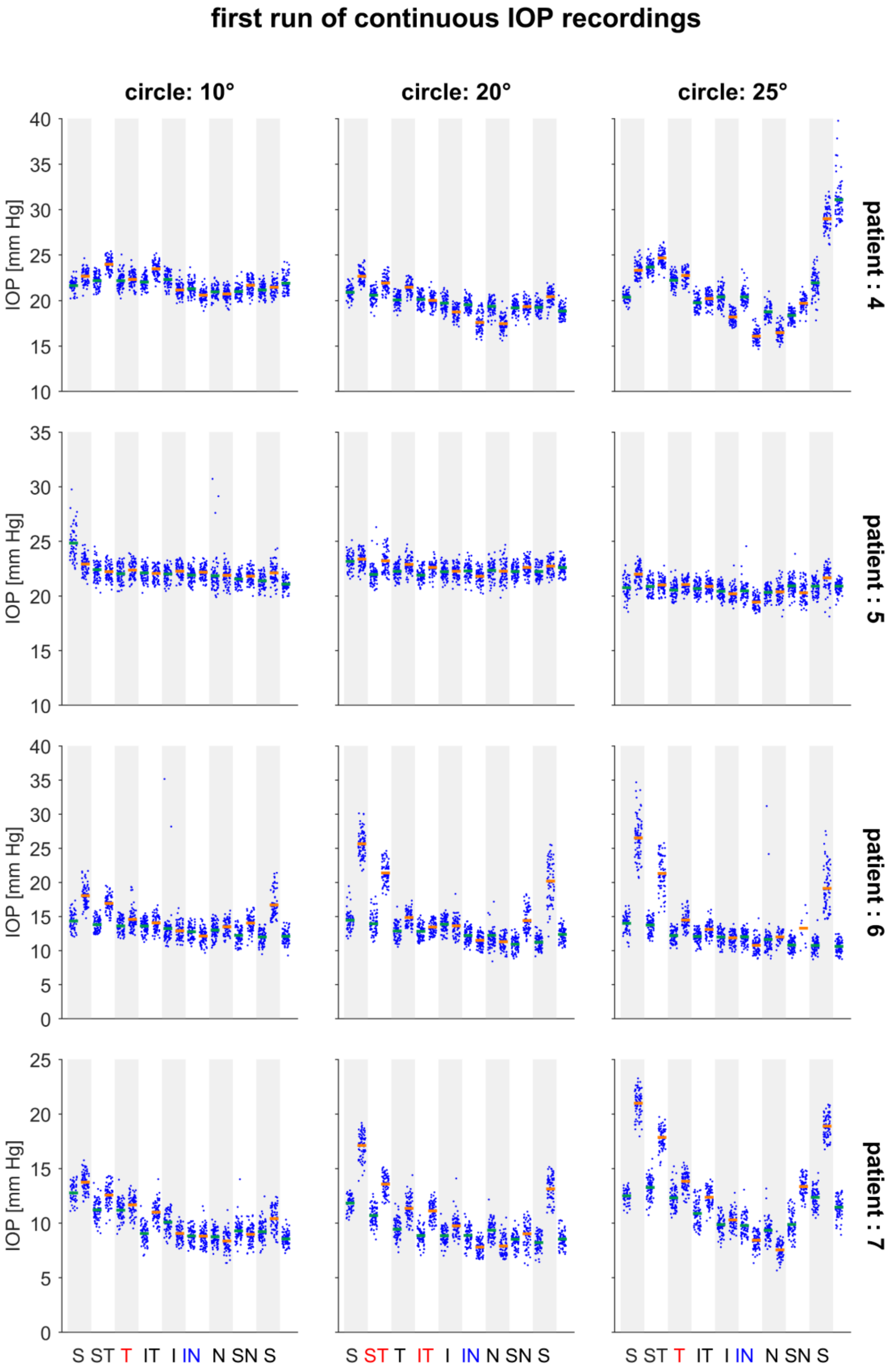
first run of continuous IOP recordings
circle: 10°
circle: 20°
circle: 25°
patient : 4
patient : 5
patient : 6
patient : 7
IOP [mm Hg]
S ST T IT I IN N SN S

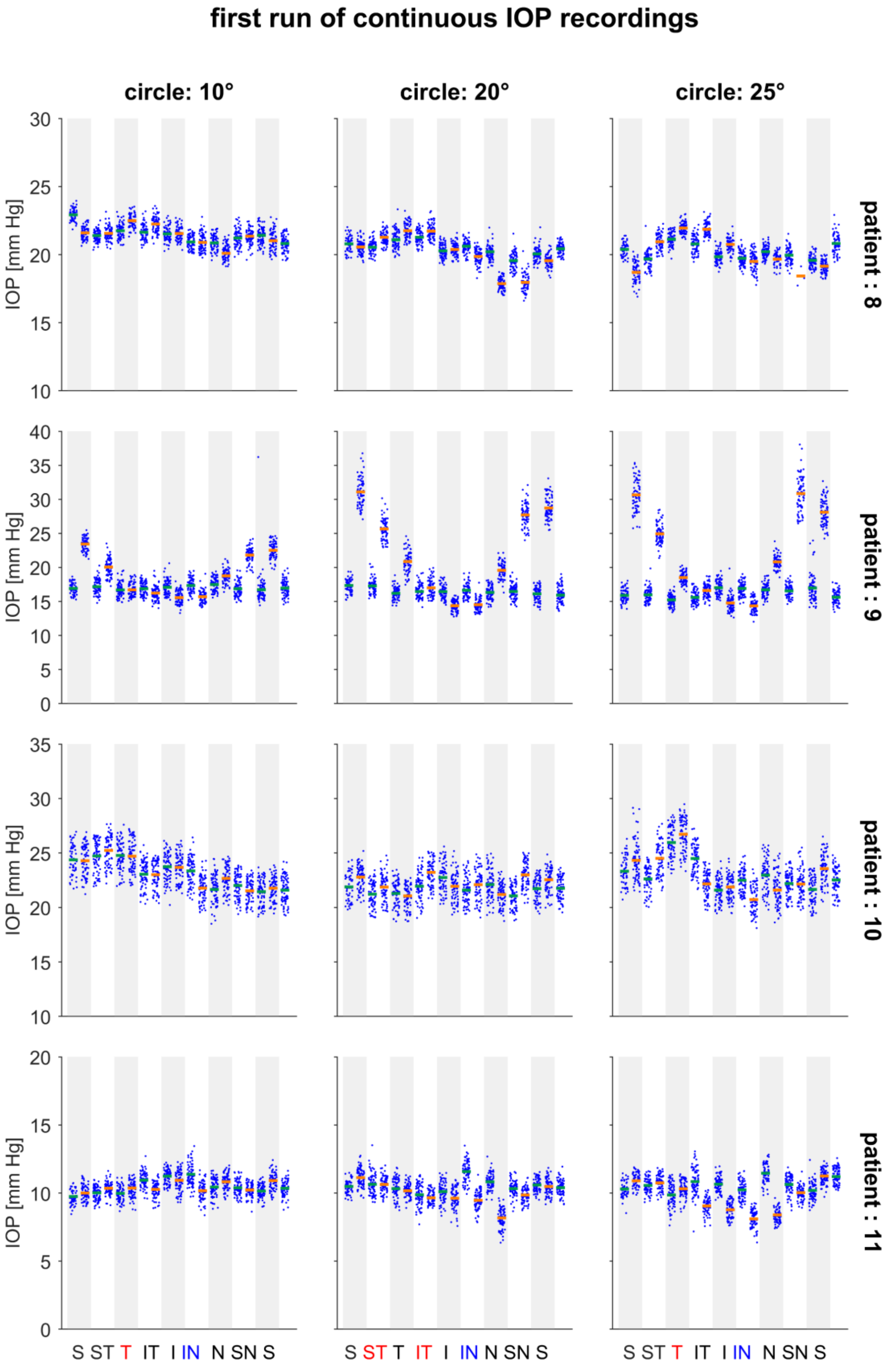

first run of continuous IOP recordings
circle: 10°
circle: 20°
circle: 25°
IOP [mm Hg]
patient : 8
patient : 9
patient : 10
patient : 11
S ST T IT I IN N SN S

Supplemental Figure 1. First run of IOP recordings of all individual patients at all three eccentricities.

Gaze directions are presented as ‘S’ (Superior), ‘ST’ (Superior Temporal), ‘T’ (Temporal), ‘IT’ (Inferior Temporal), ‘I’ (Inferior). ‘IN’ (Inferior Nasal), ‘N’ (Nasal) and ‘SN’ (Superior Nasal). Group-level significant IOP changes of eccentric gaze position compared to baseline (as shown in Table 2 and in Figure 3) are indicated in blue (decrease) and red(increase). Orange horizontal bars indicate mean IOP during primary position, green indicates mean IOP during eccentric gaze position. The first three presented patients had the sensor implant in the left eye (OS study eye). The last two periods at 25° eccentricity (second upward gaze and preceding primary position) of patient 4 were excluded from analysis due to coughing. In two patients (6 and 8), superonasal gaze position at 25° sometimes placed the sensor out of alignment with the antenna and led to a reduced number of IOP measurements.
