# Supplementary material for: Implanted Microsensor Continuous IOP Telemetry Suggests Gaze and Eyelid Closure Effects on IOP—A Preliminary Study
Source: Invest Ophthalmol Vis Sci. 2021 May 6;62(6):8. doi: 10.1167/iovs.62.6.8 (PMC8107486; doi:10.1167/iovs.62.6.8)
Supplement: Supplement 2 [file iovs-62-6-8_s002.pdf]

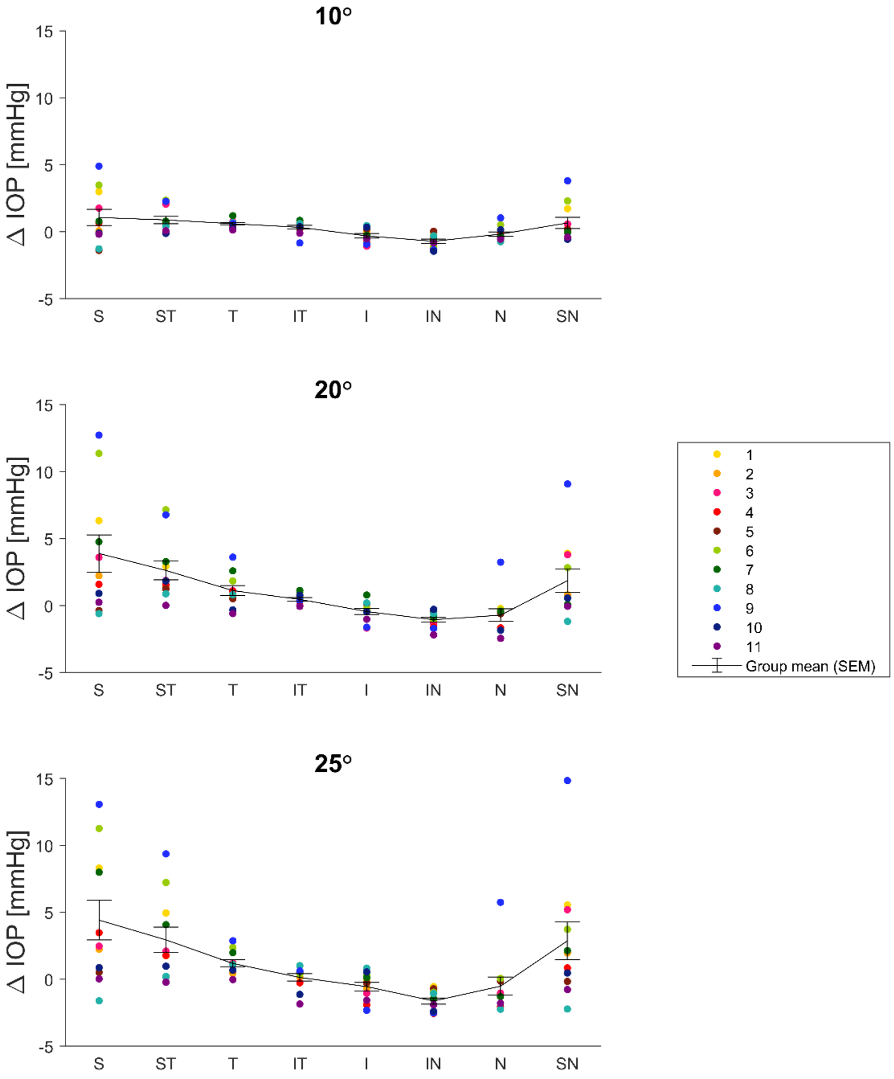

Supplemental Figure 2. Mean ΔIOP as a function of gaze direction for the three eccentricities used.

Labels indicate the four gaze directions ‘S’ (superior), ‘T’ (temporal), ‘I’ (inferior). ‘N’ (nasal). Data is also shown (without labels) for the intermediate positions (‘ST’ (superior temporal), ‘IT’ (inferior temporal), ‘IN’ (inferior nasal), ‘SN’ (superior nasal). The black lines indicate group averages and error bars (SEM), colored dots indicate individual patients (see legend). All data represent averages of 3 repetitions.
