# Supplementary material for: Implanted Microsensor Continuous IOP Telemetry Suggests Gaze and Eyelid Closure Effects on IOP—A Preliminary Study
Source: Invest Ophthalmol Vis Sci. 2021 May 6;62(6):8. doi: 10.1167/iovs.62.6.8 (PMC8107486; doi:10.1167/iovs.62.6.8)
Supplement: Supplement 3 [file iovs-62-6-8_s003.pdf]

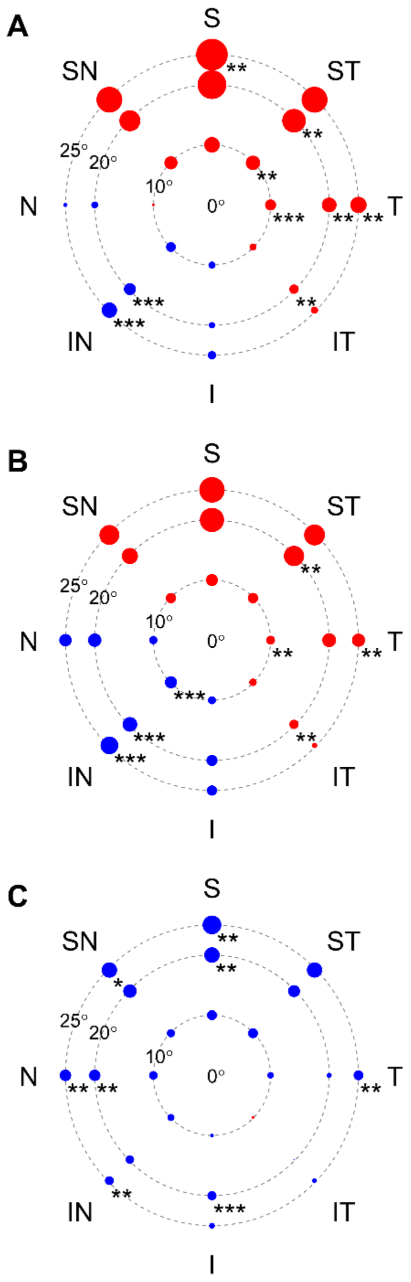

Supplemental Figure 3. Mean ΔIOP in initial ($TW_{initial}$) and final time window (TW_{final}) and the difference between them (n=11) for each gaze direction and eccentricity.

(A) mean Δ IOP during the initial 2 s at each gaze epoch ($TW_{initial}$). **(B)** mean ΔIOP during the last 2 s at each gaze epoch (TW_{final}). **(C)** difference between TW_{final} and $TW_{initial}$ (dΔIOP).

Gaze directions are presented as 'S' (Superior), 'ST' (Superior Temporal), 'T' (Temporal), 'IT' (Inferior Temporal), 'I' (Inferior). 'IN' (Inferior Nasal), 'N' (Nasal) and 'SN' (Superior Nasal). Magnitude of IOP-change scales with disc diameter. Stars indicate significance of paired T-tests corrected for multiple tests ($P \leq .01$ = **, $P \leq .001$ = ***).
