# Supplementary material for: Implanted Microsensor Continuous IOP Telemetry Suggests Gaze and Eyelid Closure Effects on IOP—A Preliminary Study
Source: Invest Ophthalmol Vis Sci. 2021 May 6;62(6):8. doi: 10.1167/iovs.62.6.8 (PMC8107486; doi:10.1167/iovs.62.6.8)
Supplement: Supplement 4 [file iovs-62-6-8_s004.pdf]

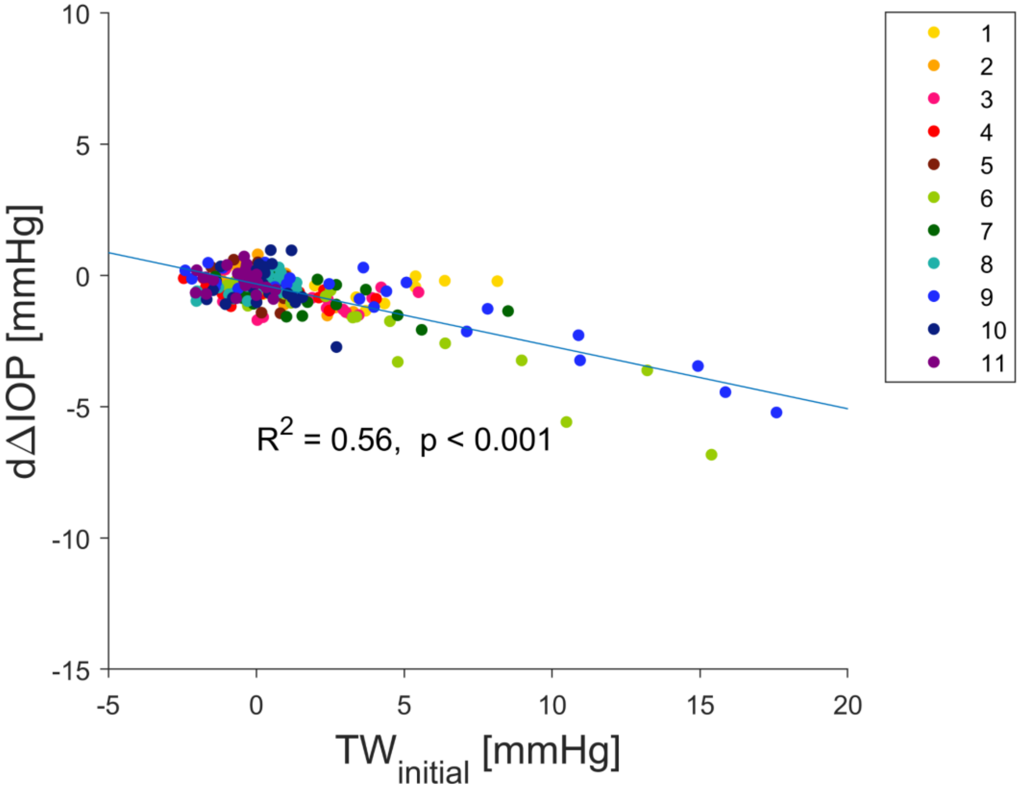

Supplemental Figure 4. Correlation of baseline IOP vs its time course within each epoch.

Correlation between $TW_{initial}$ (from baseline to beginning of new gaze position, x-axis) and dΔIOP (between $TW_{initial}$ and end of each gaze position TW_{final}, y-axis). Dots represent the 11 individual patients. R^2 correlation coefficient and P value depicted.
