# Supplementary material for: Implanted Microsensor Continuous IOP Telemetry Suggests Gaze and Eyelid Closure Effects on IOP—A Preliminary Study
Source: Invest Ophthalmol Vis Sci. 2021 May 6;62(6):8. doi: 10.1167/iovs.62.6.8 (PMC8107486; doi:10.1167/iovs.62.6.8)
Supplement: Supplement 5 [file iovs-62-6-8_s005.pdf]

Patient	ΔIOP Superior		ΔIOP Inferonasal		dΔIOP		*P*-value
	mean	SEM	mean	SEM	mean	SEM	
1	8.3	0.91	-0.5	0.34	8.9	0.59	**0.004**
2	2.2	0.87	-1.4	0.11	3.7	0.86	0.051
3	2.5	0.31	-2.4	0.10	4.9	0.26	**0.003**
4	3.5	0.29	-2.5	0.88	6.0	0.64	**0.011**
5	0.5	0.71	-0.7	0.31	1.2	1.02	0.441
6	11.3	1.06	-1.0	0.29	12.2	1.35	**0.012**
7	8.0	0.33	-1.5	0.39	9.5	0.63	**0.004**
8	-1.6	0.52	-1.1	0.42	-0.5	0.69	0.512
9	13.1	1.17	-2.5	0.15	15.6	1.08	**0.005**
10	0.9	0.15	-2.4	0.61	3.3	0.71	**0.044**
11	0.0	0.33	-1.9	0.38	1.9	0.44	**0.048**

Supplemental Table 1

Individual IOP changes during superior and inferonasal gaze direction at 25 degrees.

Normal distributed continuous data presented as mean ± SEM. Acquired IOP data during gaze experiments presented for each individual separately (N=11, mean over 3 repetitions [patient 5 performed two repetitions]). ΔIOP values shown (from left to right) for superior gaze direction, inferonasal gaze direction, and the difference between the two directions (dΔIOP). Shown *P*-values (right column) were obtained using a paired T-test within each individual (significant differences are highlighted in bold).

Marked IOP increase in upward gazes occurred in especially patients 1, 6, 7 and 9. Overall, most patients show similar trends of IOP increase or decrease in similar directions and eccentricities.
