# Supplementary material for: Implanted Microsensor Continuous IOP Telemetry Suggests Gaze and Eyelid Closure Effects on IOP—A Preliminary Study
Source: Invest Ophthalmol Vis Sci. 2021 May 6;62(6):8. doi: 10.1167/iovs.62.6.8 (PMC8107486; doi:10.1167/iovs.62.6.8)
Supplement: Supplement 6 [file iovs-62-6-8_s006.pdf]

Patient	Open eyelids		Closed eyelids		ΔIOP		*P*-value
	Mean	SEM	Mean	SEM	Mean	SEM	
1	16.0	0.15	12.6	0.11	-3.3	0.13	**0.000**
2	15.4	0.28	13.1	0.05	-2.3	0.25	**0.009**
3	15.1	0.07	11.9	0.20	-3.2	0.13	**0.000**
4	19.5	0.20	16.7	0.17	-2.8	0.12	**0.001**
6	13.7	0.23	12.3	0.30	-1.4	0.11	**0.004**
7	12.2	0.17	9.9	0.13	-2.4	0.09	**0.000**
8	19.0	0.12	17.3	0.10	-1.7	0.21	**0.013**
9	18.1	0.07	17.5	0.09	-0.6	0.07	**0.011**
10	19.2	0.36	18.4	0.49	-0.8	0.74	0.521

Supplemental Table 2

Individual IOP changes in a patient subset during eyelid closure.

Normal distributed continuous data presented as mean ± SEM. Acquired IOP data during eyelid experiments presented for each individual separately (N=9, mean over 4 repetitions). IOP values shown (from left to right) for open eyelids as baseline measurement, closed eyelids as intervention, and the difference between open eyelids and closed eyelids (ΔIOP). *P*-values (right column) were obtained using a paired T-test within each individual.
